# The Clinical Effect of Acupuncture in the Treatment of Obstructive Sleep Apnea: A Systematic Review and Meta-Analysis of Randomized Controlled Trials

**DOI:** 10.1155/2016/8792167

**Published:** 2016-04-04

**Authors:** Zheng-tao Lv, Wen-xiu Jiang, Jun-ming Huang, Jin-ming Zhang, An-min Chen

**Affiliations:** ^1^Department of Orthopedics, Tongji Hospital, Tongji Medical College, Huazhong University of Science and Technology, Wuhan, Hubei 430030, China; ^2^Department of Otolaryngology, Tongji Hospital, Tongji Medical College, Huazhong University of Science and Technology, Wuhan, Hubei 430030, China

## Abstract

*Purpose. *This study aims to determine the clinical efficacy of acupuncture therapy in the treatment of obstructive sleep apnea.* Methods. *A systematic literature search was conducted in five databases including PubMed, EMBASE, CENTRAL, Wanfang, and CNKI to identify randomized controlled trials (RCTs) on the effect of acupuncture therapy for obstructive sleep apnea. Meta-analysis was conducted using the RevMan version 5.3 software.* Results. *Six RCTs involving 362 subjects were included in our study. Compared with control groups, manual acupuncture (MA) was more effective in the improvement of apnea/hypopnea index (AHI), apnea index, hypopnea index, and mean SaO_2_. Electroacupuncture (EA) was better in improving the AHI and apnea index when compared with control treatment, but no statistically significant differences in hypopnea index and mean SaO_2_ were found. In the comparison of MA and nasal continuous positive airway pressure, the results favored MA in the improvement of AHI; there was no statistical difference in the improvement in mean SaO_2_. No adverse events associated with acupuncture therapy were documented.* Conclusion. *Compared to control groups, both MA and EA were more effective in improving AHI and mean SaO_2_. In addition, MA could further improve apnea index and hypopnea index compared to control.

## 1. Introduction

Obstructive sleep apnea (OSA) is a major public health issue affecting children and adults which is characterized by reduced airflow during sleep resulting in gas exchange abnormalities and disrupted sleep [1]. The pathogenesis of OSA is complicated but it is probably due to a combination of an anatomically small pharyngeal airway in conjunction with a sleep related decline in upper airway dilator muscle activity [2, 3]. OSA occurs more commonly in men than in women, and predisposing risk factors include obesity, adenotonsillar hypertrophy, retrognathia, hypothyroidism, nasal obstruction, and evening alcohol ingestion [4]. Patients with OSA exhibit reduced quality of life due to daytime symptoms such as excessive sleepiness, irritability, decreased concentration and memory, reduced energy, erectile dysfunction, depressive symptoms, and an association with cardiovascular and metabolic diseases that restrict their social activities [5–12].

The gold standard for documenting severity of OSAS is overnight polysomnography (PSG). Considering the socioeconomic burden of OSA, patients with OSA should be treated immediately after diagnosis. In view of the high prevalence and the relevant impairment of patients with OSA, lots of methods are offered for the improvement of OSA. Nasal continuous positive airway pressure (nCPAP) therapy is accepted as the standard treatment for the management of clinically significant OSA in recent decades [13]. Proper use of nCPAP manages apnea and hypopnea, eliminates hypoxia, restores normal sleep architecture, and significantly improves subjective and objective measures of wakefulness and averts cardiovascular consequences, especially arterial hypertension [14]. In addition to nCPAP, oral appliances may be considered as a long-term alternative in patients with severe OSAS who do not respond to CPAP or in whom treatment attempts with CPAP fail. Surgery may also be recommended with curative intent for patients with an obvious anatomic obstruction such as large palatine or lingual tonsils or used as a salvage procedure to improve OSA in patients who fail CPAP and/or other treatment measures [15, 16].

The standard treatment, nCPAP, has been proven to reduce upper airway obstructions and improves quality of life. Despite the notable efficacy of nCPAP, many patients suffer from local side effects at the nose or face or discomfort due to the mask [17]. Moreover, CPAP does not allow for a permanent resolution of respiratory disturbances during sleep but only suppresses them while using the devices [17, 18]. Patients often have difficulty in adhering to nCPAP or may switch to complementary and alternative (CAM) therapy [19]. Those with OSA who choose CAM approaches are potentially seeking ways to improve chronic fatigue and fragmented sleep. As a mainstream of CAM therapy, acupuncture has been practiced for thousands of years in China for the treatment of various diseases [20]. Given the lack of now-existing evidence showing the beneficial effect of CAM therapies, they cannot be recommended as a primary treatment of OSA. It seems that there are no alternatives to the conventional treatment of OSAS which provide the same positive outcomes as CPAP, surgical interventions, or oral appliances when used appropriately for selected patients [1]. Thus, the aim of our present work was to evaluate the clinical effect of acupuncture therapy in the treatment of OSA, which could be an affordable treatment for OSA.

## 2. Materials and Methods

This systematic review and meta-analysis was performed strictly in accordance with the Preferred Reporting Items for Systematic Reviews and Meta-Analyses (PRISMA) guidelines [21].

### 2.1. Search Strategy

A systematic literature search was conducted using the following electronic databases: Pubmed, EMBASE, CENTRAL, Wanfang, and CNKI. All these electronic databases were searched from their inception dates up to the latest issue (October 2015). The bibliographies of relevant systematic reviews and clinical guidelines were manually searched; no language restriction was imposed. A combination of medical subject headings (MeSH) and free terms was applied to retrieve the potentially eligible studies as possible; MeSH was slightly modified based on the specification of each database.

The search terms of English databases were as follows: (“Sleep Apnea, Obstructive” or osahs OR obstructive sleep apnea OR sleep apnea OR sleep hypopnea OR upper airway resistance sleep apnea syndrome) and (“Acupuncture Therapy” or acupuncture or moxibustion or acupoint or acupressure OR acustimulation); for Chinese databases we used search terms as “zhen” and (“shuimian” and (“huxizanting” or “ditongqi” or “zusexing”)). The detailed procedure of literature search in Pubmed and EMBASE was presented in Appendix.

### 2.2. Inclusion and Exclusion Criteria

The PICOS (participants, interventions, comparisons, outcomes, and study design) principle was utilized for our inclusion and exclusion criteria.

Participants included in our study had to be diagnosed with OSA according to the results of PSG (AHI > 5). No restrictions on age, sex, and race were imposed. Patients with OSA in the experimental groups mainly received acupuncture therapy including manual acupuncture (MA) and electroacupuncture (EA), without differentiating the needle materials and acupoints selection; subjects allocated in the control groups received no specific treatment or sham acupuncture (SA) or nCPAP treatment. The primary outcome was apnea/hypopnea index (AHI) and the second outcomes included hypopnea index, apnea index, and mean SaO_2_. To be included in our current review, the study design had to be randomized controlled trial. Animal experiments, review, case report, and studies that were duplicates for retrieving or publishing were excluded.

### 2.3. Data Extraction

Two independent reviewers (Zheng-tao Lv and Wen-xiu Jiang) reviewed each article and each one of them was blinded to the findings of the other. Raw data was independently extracted and collected from the original articles by two reviewers; data extraction was guided by a predetermined standardized collection form which includes first author and year, country, study design, baseline characteristics of participants, diagnostic criteria for OSA, interventions in experimental and control groups, duration of treatment, and main outcome assessments. Any discrepancies between reviewers were resolved through discussion until a consensus was reached. A third author (An-min Chen) was consulted if a consensus could not be reached.

### 2.4. Risk of Bias Assessment

To assess the methodological quality of selected studies, Cochrane Collaboration's tool [28] was used, which was based on seven items: random sequence generation, allocation concealment, blinding of participants and personnel, blinding of outcome assessment, incomplete outcome data, selective reporting, and other sources of bias. The response for each criterion was reported as low risk of bias, high risk of bias, and unclear risk of bias. Two reviewers evaluated the quality of trials independently.

### 2.5. Data Synthesis and Analysis

The meta-analysis and statistical analyses were performed using the RevMan 5.3 analyses software of the Cochrane Collaboration. Since the types of all the outcome measurements were continuous variables, mean differences (MD) and the associated 95% confidence interval (CI) were calculated for AHI, hypopnea index, apnea index, and mean SaO_2_. Heterogeneity among studies was assessed using Chi-squared test and Higgins *I* ^2^ test (*I* ^2^ < 50% indicates acceptable heterogeneity); we pooled data across studies using random effect model if obvious heterogeneity existed; otherwise, a fixed effect model would be used. In case of obvious heterogeneity, subgroup analysis was conducted based on the specification of acupuncture techniques. Publication bias was detected via a funnel plot if the amount of included studies was greater than 10.

## 3. Results

### 3.1. Literature Search

**Figure 1:**
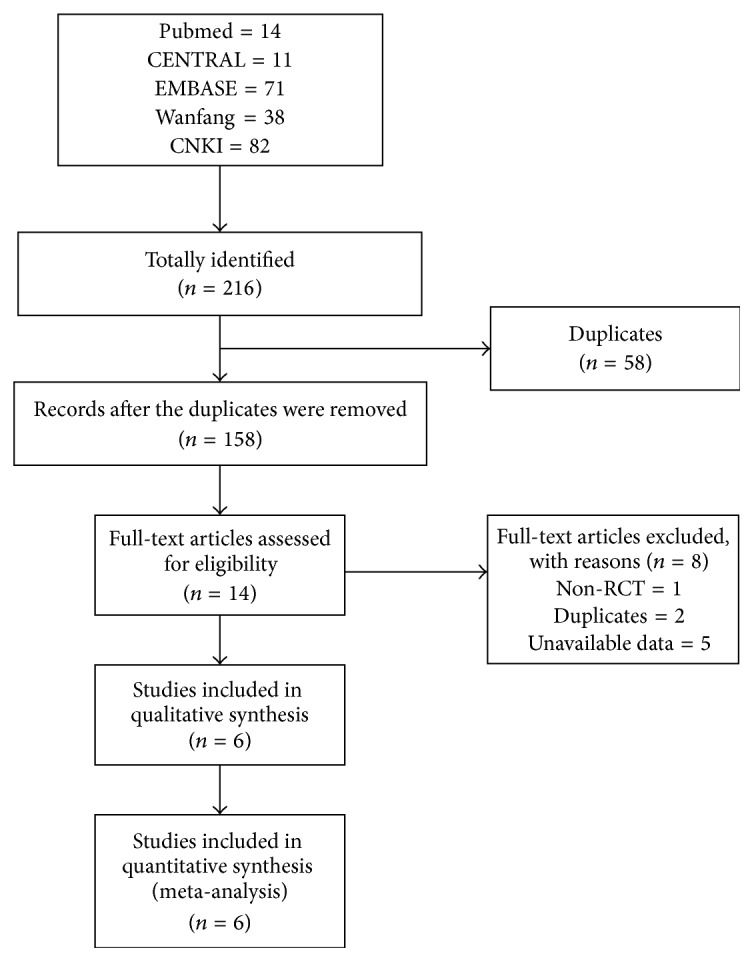
Flowchart of the literature search.

The literature screening process is presented in Figure 1. An initial search yielded 216 potential literature citations, including 14 records from Pubmed, 11 from CENTRAL, 71 from EMBASE, 38 from Wanfang, and 82 from CNKI. 58 records were excluded because they were duplicates. 158 studies were considered potentially eligible by reading their titles and abstracts. According to the predetermined inclusion criteria, 14 articles remained to be evaluated using a full-text screen. Of the remaining 14 studies, one study was excluded because it was not RCT, two studies were excluded because they were duplicates, and five studies were excluded because of unavailable data reported. Finally, six studies [22–27] were deemed eligible to be included in our meta-analysis.

### 3.2. The Characteristics of Included Trials

The basic demographic information and detailed intervention methods are listed in Tables 1 and 2. Two studies [22, 24] were conducted in Brazil and the other four [23, 25–27] were conducted by Chinese investigators; each study was performed at a single center. These RCTs were published between 2007 and 2015; a total of 362 patients were enrolled: 197 patients in the acupuncture group and 165 patients in control group. Age of the participants ranged from 35 to 76; baseline similarities were reported in each study. All the studies conducted in China used a two-arm parallel design, two studies [25, 27] were designed to evaluate the clinical effect of EA compared to nonspecific treatment, and the other two studies [23, 26] aimed to compare the efficacy of MA and that of nCPAP. The single-blinded RCT [22] conducted in 2007 used a three-arm parallel design; MA was compared with no treatment and SA. A four-arm parallel RCT [24] was conducted by Freire and colleagues in 2010, the clinical efficacy of MA and EA with different power frequencies was compared with that of control group, and parameters associated with OSA (AHI, apnea index, hypopnea index, and mean SaO_2_) were assessed by PSG.

### 3.3. Risk of Bias Assessment

**Figure 2:**
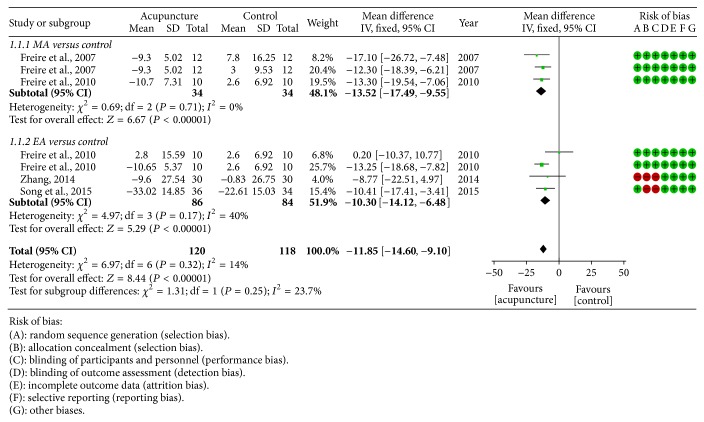
Forest plot of acupuncture therapy versus control group: AHI; the authors' judgment about each risk of bias item for each included study.

**Figure 3:**
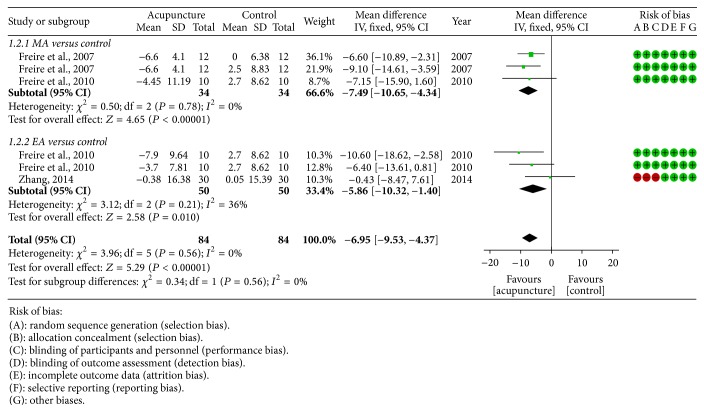
Forest plot of acupuncture therapy versus control group: apnea index; the authors' judgment about each risk of bias item for each included study.

**Figure 4:**
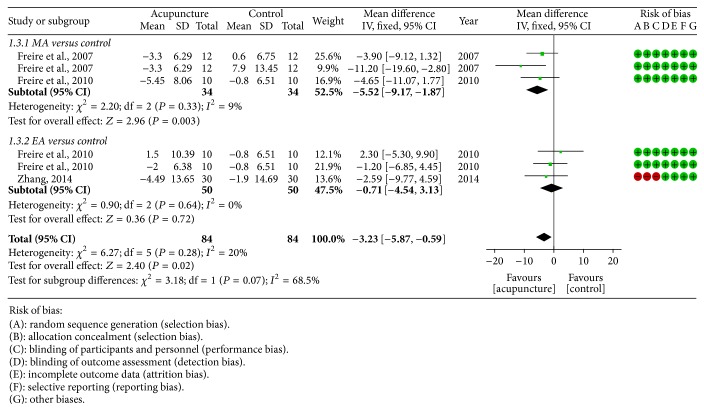
Forest plot of acupuncture therapy versus control group: hypopnea index; the authors' judgment about each risk of bias item for each included study.

**Figure 5:**
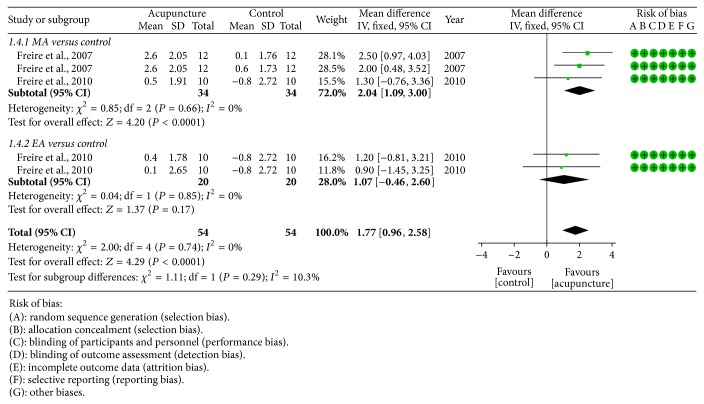
Forest plot of acupuncture therapy versus control group: mean SaO_2_; the authors' judgment about each risk of bias item for each included study.

**Figure 6:**
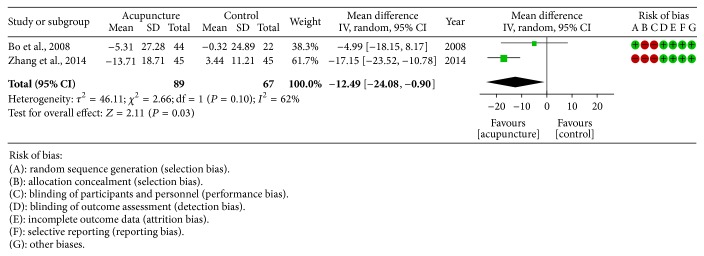
Forest plot of MA versus nCPAP: AHI; the authors' judgment about each risk of bias item for each included study.

**Figure 7:**
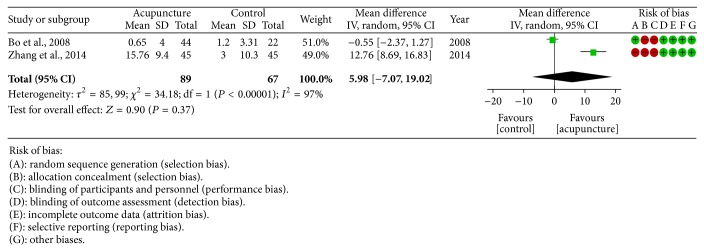
Forest plot of MA versus nCPAP: mean SaO_2_; the authors' judgment about each risk of bias item for each included study.

To assess the risk of bias among included studies, Cochrane Collaboration's tool was employed. All of the six studies reported suggested randomization; however, two studies [25, 26] failed to provide the method of random sequence generation. Only two studies [22, 24] reported the procedure of allocation concealment, and the blinding of participants and personnel was carried out appropriately in these two trials; the investigators conducted RCT according to a strict study protocol approved by the ethical committee of the Universidade Federal de Sao Paulo. None of the four remaining studies [23, 25–27] provided detailed information about the allocation concealment and blinding of participants and personnel. The blinding of outcome measure was judged to low risk of bias because all the outcomes were measured depending on the records of PSG; the accuracy and objectivity were unlikely to be influenced by lack of blinding. Regarding the selective reporting, all the trials were judged to low risk of bias, since we only included studies that reported AHI, apnea index, hypopnea index, and mean SaO_2_ as outcome. No study reported adverse events associated with acupuncture sessions. Good compliance seemed to be achieved in all studies; each study reported characterized similarity of baseline. Finally, two studies [22, 24] were judged to low risk of bias; the four remaining studies [23, 25–27] were judged to high risk of bias. The risk of bias assessment of each study was listed in corresponding forest plot (Figures 2, 3, 4, 5, 6, and 7).

### 3.4. Meta-Analysis Results

#### 3.4.1. Acupuncture versus Control

*AHI.* Four studies [22, 24, 25, 27] measured AHI as outcome; a fixed effect model was employed because there was no obvious heterogeneity among included studies. Compared with control group, both MA (−13.52 [−17.49, −9.55]) and EA (−10.30 [−14.12, −6.48]) could further improve AHI (Figure 2).

*Apnea Index.* Three studies [22, 24, 25] measure apnea index as outcome measurement; fixed effect model was used, since there was no obvious heterogeneity among the included studies. Compared with control group, both MA (−7.49 [−10.65, −4.34]) and EA (−5.86 [−10.32, −1.40]) could further improve apnea index (Figure 3).

*Hypopnea Index.* Three studies [22, 24, 25] measured hypopnea index as outcome measurement. Fixed effect model was used for statistical analysis because there was no obvious heterogeneity among studies. The pooled data showed that MA was more effective in the improvement of hypopnea index compared with control group (−5.52 [−9.17, −1.87]), whereas there was no significant difference between EA (−0.71 [−4.54, 3.13]) and control group (Figure 4).

*Mean SaO*_*2*_. Two studies [22, 24] measured mean SaO_2_ as outcome assessment. Since there was no obvious heterogeneity among studies, fixed effect model was utilized for statistical analysis. The combined data suggested that MA (2.04 [1.09, 3.00]) could further improve mean SaO_2_ but EA (1.07 [−0.46, 2.60]) could not (Figure 5).

#### 3.4.2. Acupuncture versus nCPAP

*AHI*. Two studies [23, 26] employed AHI as outcome; obvious heterogeneity existed among studies (heterogeneity: *τ* ^2^ = 46.11; *χ* ^2^ = 2.66; df = 1 (*P* = 0.10); *I* ^2^ = 62%). Thus, random effect model was utilized for data analysis; the combined data showed that MA was more effective in improving the AHI when compared with nCPAP (−12.49 [−24.08, −0.90]) (Figure 6).

*Mean SaO*_*2*_. Two studies [23, 26] recorded mean SaO_2_ in MA group and nCPAP group; the heterogeneity could be observed so the random effect model was used (heterogeneity: *τ* ^2^ = 85.99; *χ* ^2^ = 34.18; df = 1 (*P* < 0.00001);* I* ^2^ = 97%). Regarding the improvement in mean SaO_2_ no significant difference could be detected between MA and nCPAP (5.98 [−7.07, 19.02]) (Figure 7).

### 3.5. Adverse Events

All the enrolled patients were informed about the possible risks of acupuncture treatment such as infection, fainting, and hematoma. Ideal compliance seemed to be achieved in each study; no adverse events associated with acupuncture therapy were reported.

### 3.6. Publication Bias

The publication bias in our meta-analysis was not explored since the amount of included studies was insufficient. The potential of publication bias could not be excluded.

## 4. Discussion

To the best of our knowledge, this is the first meta-analysis aiming to assess the clinical effect of acupuncture therapy in the treatment of OSA; six studies involving 362 subjects were selected in our study. The findings of our work suggest that MA was more effective in the improvement of AHI, apnea index, hypopnea index, and mean SaO_2_ when compared with nonspecific treatment; EA could further improve AHI and apnea index; there was no significant difference regarding the improvement of hypopnea index and SaO_2_. Regarding the comparison of MA and nCPAP, MA was more effective in improving AHI. No adverse events associated with acupuncture therapy were documented.

The goal of OSA treatment is reduction in sleep disruption and the AHI, with resultant improved overall health and quality of life. Despite the remarkable efficacy of nCPAP, patients often have difficulty in adhering to it or may switch to CAM therapy because of the cumbersome nature of CPAP and the socioeconomic burden. In our current review, acupuncture therapy was compared with nonspecific treatment and nCPAP separately. In the comparison of MA and control group, all included studies showed a consistency regarding the improvement of AHI, apnea index, hypopnea index, and mean SaO_2_; heterogeneity among these studies was acceptable. However, compared to control group, EA was only effective in the improvement of AHI and apnea index. In terms of the comparison between MA and nCPAP, MA was more effective in the improvement of AHI; no significant difference was found in the improvement of SaO_2_.

Based on the quality assessment of our included studies, only two studies were judged to low risk of bias, whereas the remaining four studies were judged to high risk of bias. The methodological deficiency might limit the paucity of conclusions and lead to overstatement of clinical efficacy of acupuncture therapy. Lack of blinding procedures in RCTs can also exaggerate the conclusions of these trials. In the clinical trial conducted by Ernest and Resch, specific and/or nonspecific effects indicated that a treatment had been successful [29]. Acupuncture has the potential to elicit very powerful placebo effects [30]. Not surprisingly, therefore, almost all patients treated with sham acupuncture may respond positively in some manner [31]. In our study, we selected AHI, apnea index, hypopnea index, and mean SaO_2_ as outcome assessment because these data could be directly recorded by overnight PSG. Thus, the accuracy and the objectivity of outcome would not be influenced by lack of blinding.

As an alternative modality of MA, EA has been used frequently in clinical and basic search, but the underlying mechanism of EA and MA might differ to some extent, since EA causes the release of beta-endorphin and adrenocorticotrophic hormone into plasma, whereas MA releases only beta-endorphin [20, 32]. Freire et al. found that comparison of the results between the groups after treatment showed that the MA group and the 10 Hz EA group significantly differed from both the 2 Hz EA and control groups in all the polysomnographic parameters, specifically in the primary outcome, AHI. Freire and colleagues attributed this improvement to the involvement of serotonergic pathways in the responses mediated by acupuncture as well as its anti-inflammatory effect [33–35]. In our systematic review, different types of acupuncture including MA, 2 Hz EA, and 10 Hz EA were treated as one type of therapy and the data were combined without differentiating acupoint selection or acupuncture modalities. Thus, the findings of this review indicate an overall trend of efficacy; definite conclusions could not be drawn.

Proper ethical research needs to take into consideration not only the cost of treatment or wait time for treatment but also a thorough understanding of the nature of acupuncture therapy. Further studies with strict study design and larger sample size are encouraged.

## 5. Conclusion

In summary, the results of our review suggest that both MA and EA were effective in improving AHI and mean SaO_2_; additionally, MA could further improve apnea index and hypopnea index when compared with control treatment. Regarding the comparison of MA and nCPAP, no definite conclusion could be drawn due to the limited evidence. Additional RCTs with rigorous study design and larger sample size are required.

## Figures and Tables

**Table 1:** Characteristics of included studies.

Study	Country	Study design	Population	Age (mean or range)	EC approval
Freire et al., 2007 [22]	Brazil	RCT	MA: 12 SA: 12 Control: 12	MA: 54.0 (51.0–63.0) SA: 53.0 (49.0–63.0) Control: 57.0 (50.0–64.0)	Yes

Chen et al. 2008 [23]	China	RCT	MA: 44 nCPAP: 22	MA: 55.44 ± 11.04 nCPAP: 56.73 ± 10.21	Not reported

Freire et al., 2010 [24]	Brazil	RCT	MA: 10 2 Hz EA: 10 10 Hz EA: 10 Control: 10	MA: 57.7 (44.0–68.0) 2 Hz EA: 52.9 (33.0–69.0) 10 Hz EA: 54.8 (35.0–71.0) Control: 54.3 (35.0–69.0)	Yes

Zhang, 2014 [25]	China	RCT	2 Hz EA: 30 Control: 30	2 Hz EA: 69.45 ± 6.78 Control: 70.01 ± 5.94	Not reported

Zhang et al. 2014 [26]	China	RCT	MA: 45 nCPAP: 45	MA: 48.45 ± 9.76 nCPAP: 51.96 ± 9.87	Not reported

Song et al., 2015 [27]	China	RCT	2 Hz EA + nCPAP: 36 nCPAP: 34	2 Hz EA + nCPAP: 53.17 ± 10.20 nCPAP: 52.71 ± 11.26	Yes

*Note*. RCT: randomized controlled trial; MA: manual acupuncture; SA: sham acupuncture; EA: electroacupuncture; nCPAP: nasal continuous positive airway pressure; EC: ethical committee.

**Table 2:** Interventions and outcome assessment of included studies.

Study	Diagnostic criteria for OSAHS	Duration of treatment	Experimental treatment	Control treatment	Main outcome
Freire et al., 2007 [22]	PSG 15 < AHI < 30	10 weeks	MA: (Gv20, Li20, Ren23, P6, Lu7, Li4, St36, St40, Sp6, Kd6) 30 min, deqi	Control: weight reduction advice and sleep hygiene counseling SA: (acupoints were 1 cun from the real point) 30 min, no manipulation	AHI, AI, HI, mean SaO_2_

Chen et al., 2008 [23]	PSG AHI > 5	20 days	MA: (Cv23, Panglianquan, Si17, L7, K6, Sp4, Cv17, S40, H7, Sp6, extra6) 30 min, deqi	nCPAP: once a day, 20 days in total	AHI, AI, HI, mean SaO_2_

Freire et al., 2010 [24]	PSG 15 < AHI < 30	1 night	MA: (Lu6, Lu7, Li4, Li20, Gv20, Cv23, St36, St40, Sp6, Ki6, Extra12) 30 min, deqi 2 Hz EA: (Cv23, Extra12, Li4, St36) 30 min, 0.6–0.8 mA, 2 Hz 10 Hz EA: (Cv23, Extra12, Li4, St36) 30 min, 0.6–0.8 mA, 10 Hz	No specific treatment reported	AHI, AI, HI, mean SaO_2_

Zhang, 2014 [25]	PSG AHI > 5	20 days	2 Hz EA: (Cv23, Panglianquan) once a day	No specific treatment reported	AHI, AI, HI

Zhang et al. 2014 [26]	PSG AHI > 5	4 weeks	MA: (Li11, S25, Sp9, S40, Liv3) 30 min, deqi; weight reduction advice and smoking cessation	nCPAP: details are not reported; weight reduction advice and smoking cessation	AHI, mean SaO_2_

Song et al., 2015 [27]	PSG AHI > 15	6 weeks	nCPAP + 2 Hz EA: (Extra8, Extra9, Extra6, H7, St36, Sp6, K6) 2 Hz, 30 min	nCPAP: 3 times a week, 6 weeks in total	AHI

*Note*. MA: manual acupuncture; SA: sham acupuncture; EA: electroacupuncture; nCPAP: nasal continuous positive airway pressure; PSG: polysomnography; AHI: apnea/hypopnea index; HI: hypopnea index; AI: apnea index.

## Appendix

*Pubmed*

1. “Sleep Apnea, Obstructive” [MeSH]
2. (OSAHS OR obstructive sleep apnea OR sleep apnea OR sleep hypopnea OR upper airway resistance sleep apnea syndrome)
3. (1) or (2)
4. “Acupuncture Therapy” [MeSH]
5. (acupuncture or moxibustion or acupoint or acupressure OR acustimulation)
6. (4) or (5)
7. (3) and (6)

*EMBASE*

(1) exp acupuncture/
(2) (acupuncture or acupuncture therapy or meridians or moxibustion or ear acupuncture).af.
(3) (1) or (2)
(4) exp sleep disordered breathing/
(5) (Obstructive sleep apnea^*∗*^ or obstructive sleep apnea hypopnea syndrome or sleep apnea^*∗*^).af.
(6) (4) or (5)
(7) (3) and (6)

## Additional Points

This review highlights the clinical effect of manual acupuncture and electroacupuncture in the treatment of patients with obstructive sleep apnea.
